# Internal drainage for interdisciplinary management of anastomotic leakage after pancreaticogastrostomy

**DOI:** 10.1007/s00464-023-09964-1

**Published:** 2023-03-06

**Authors:** Matthäus Felsenstein, Ann-Christin Amini, Sophie Dorfer, Mengwen Hu, Ruonan Wang, Lea Timmermann, Karl Herbert Hillebrandt, Christian Benzing, Uli Fehrenbach, Uwe Pelzer, Igor Maximillian Sauer, Johann Pratschke, Christian Jürgensen, Thomas Malinka

**Affiliations:** 1 Department of Surgery, CCM | CVK, Charité–Universitätsmedizin Berlin, Corporate Member of Freie Universität Berlin, Humboldt-Universität Zu Berlin, and Berlin Institute of Health, 13353 Berlin, Germany; 2 Berlin Institute of Health (BIH), Charité–Universitätsmedizin Berlin, Berlin, Germany; 3 Department of Radiology, CCM | CVK, Charité–Universitätsmedizin Berlin, Berlin, Germany; 4 Division of Hematology, Oncology and Tumor Immunology, Medical Department, CCM, Charité–Universitätsmedizin Berlin, Berlin, Germany; 5 Department of Hepatology and Gastroenterology, CCM | CVK, Charité–Universitätsmedizin Berlin, Berlin, Germany

**Keywords:** Endoscopy-guided drainage, Peri-anastomotic stent, Intramural drainage, Anastomotic leakage, Pancreatic fistula

## Abstract

**Background:**

Anastomotic leakage and postoperative pancreatic fistula (POPF) may occur after pancreatic head resection, also in the setting of pancreato-gastric reconstruction. For adequate complication management, a variety of non-standardized treatments are available. Still, data on clinical evaluation of endoscopic methods remain scarce. Based on our interdisciplinary experience on endoscopic treatment of retro-gastric fluid collections after left-sided pancreatectomies, we developed an innovative endoscopic concept with internal peri-anastomotic stent placement for patients with anastomotic leakage and/or peri-anastomotic fluid collection.

**Methods:**

Over the period of 6 years (2015–2020) we retrospectively evaluated 531 patients after pancreatic head resections at the Department of Surgery, Charité–Unversitätsmedizin Berlin. Of these, 403 received reconstruction via pancreatogastrostomy. We identified 110 patients (27.3%) with anastomotic leakage and/or peri-anastomotic fluid collection and could define four treatment groups which received either conservative treatment (C), percutaneous drainage (PD), endoscopic drainage (ED), and/or re-operation (OP). Patients were grouped in a step-up approach for descriptive analyses and in a stratified, decision-based algorithm for comparative analyses. The study’s primary endpoints were hospitalization (length of hospital stay) and clinical success (treatment success rate, primary/secondary resolution).

**Results:**

We characterized an institutional, post-operative cohort with heterogenous complication management following pancreato-gastric reconstruction. The majority of patients needed interventional treatments (*n* = 92, 83.6%). Of these, close to one-third (*n* = 32, 29.1%) were treated with endoscopy-guided, peri-anastomotic pigtail stents for internal drainage as either primary, secondary and/or tertiary treatment modality. Following a decision-based algorithm, we could discriminate superior primary—(77,8% vs 53.7%) and secondary success rates (85.7% vs 68.4%) as well as earlier primary resolutions (11.4 days, 95%CI (5.75–17.13) vs 37.4 days, 95%CI (27.2–47.5)] in patients receiving an endoscopic compared to percutaneous management.

**Conclusion:**

This study underscores the importance of endoscopy-guided approaches for adequate treatment of anastomotic leakage and/or peri-anastomotic fluid collections after pancreatoduodenectomy. We herein report a novel, interdisciplinary concept for internal drainage in the setting of pancreato-gastric reconstruction.

Anastomotic leakage after pancreatoduodenectomy remains a technical bottleneck resulting in increased morbidity and mortality [1]. Various treatment modalities are widely used to address intraabdominal fluid collections and fistulas after pancreatic surgery [2]. Following pancreato-enteric reconstruction, anastomotic leakage can be a significant source of pancreatic fistulas and peri-anastomotic fluid collection. This regularly demands immediate intervention due to potential hazardous complications such as hemorrhages and sepsis [3]. For complication management commonly considers conservative (antibiotics, octreotide, parental nutrition, irrigation of intraoperative drains), interventional (percutaneous drain, endoscopic drain), and/or surgical treatments in step-up strategies [2, 4, 5].

However, a combination of treatment modalities is regularly needed for complete resolution culminating in prolonged hospitalization and delayed re-convalescence [6]. Previous studies demonstrated the advantages of endoscopic management for peri-gastric fluid collections after distal pancreatectomy [7, 8]. This has been further translated to other indications while few studies included patients after pancreatoduodenectomy [9–12]. Initially, Tilara et al. described clinical success with trans-gastric stents for treatment of peri-gastric fluid collections early after pancreaticoduodenectomy [9]. A subsequent study by Al-Efishat M et al*.* were able to demonstrate comparable technical and clinical success rates of endoscopy-guided, transmural stent placement over percutaneous drainage in a matched-pair analyses [11]. Still, studies on pancreatic fistula with fluid collections after pancreato-enteric reconstruction remain scarce. Therefore, many pancreatic centers primarily rely on percutaneous and operative interventions which challenges the implementation of modern enhanced recovery programs (ERAS) for pancreatic procedures [13, 14].

Our institutional experience treating peri-gastric fluid collections and pancreatic fistula after distal pancreatectomies has helped us to further advance on interventional techniques [15]. For reconstruction after pancreatoduodenectomy, we regularly perform a refined technique of pancreatogastrostomy in both, open and robotic procedures [16, 17]. Still, anastomotic leakage occurs during the postoperative course but can now be directly addressed by endoscopic intervention. In cases of confirmed anastomotic leakage, we regularly perform endoscopy-guided, peri-anastomotic stent placement for internal drainage of peri-anastomotic fluid collection. Positive experiences with early endoscopic intervention on selected patients created some optimism about effectively decreasing hospitalization and morbidity. We sought to retrospectively analyze the safety and efficacy of our novel treatment modality following pancreatoduodenectomy with pancreato-gastric reconstruction.

## Methods

### Patients

The study enrolled all patients that underwent open or minimal-invasive (robotic) pancreatoduodenectomy with pancreato-gastric reconstruction at Charité–Universitätsmedizin Berlin from January 2015 to December 2020 (*n* = 403). We selected patients fulfilling radiographic (CT-scan) criteria for peri-anastomotic fluid collection or endoscopic assessment of anastomotic leakage (*n* = 110). At our institution, treatments normally started only after one of these diagnostic modalities was conducted. Therefore, patients showing biochemical leakage (POPF A) without detection of fluid collection in CT-scan or dehiscence during endoscopy were not included in the study. We retrospectively analyzed the clinical course and complication management in a prospectively collected database. Demographics and baseline characteristics, including age, sex, surgery technique, pre-/peri-operative information, as well as histopathologic diagnoses were documented. All patients gave their informed consent for statistical evaluation of their clinical course after pancreatic surgery (Approval Institutional ethics board EA1/341/20).

### Definition of treatment groups

The complication management during the study period after diagnosis of peri-anastomotic fluid collection and/or anastomotic leakage included four main treatment modalities:

1. Conservative (C)–Treatment with only antibiotics, somatostatin analogues and/or irrigation of intraoperative drainages
2. Percutaneous drainage (PD)–Treatment with CT-guided drainage placement
3. Endoscopic drainage (ED)–Placement of endoscopy-guided internal pigtail stents
4. Re-operation (OP)–Treatment with re-operation

For statistical evaluation, the treatment groups were generated based on two distinct concepts:

#### Cohort characterization (results “Cohort characterization and Treatment course of patients with anastomotic leakage after pancreatogastrostomy”)

Patients were grouped in a step-up approach for descriptive analyses, meaning that patients were allocated according to their most invasive procedure for treatment of anastomotic leakage:

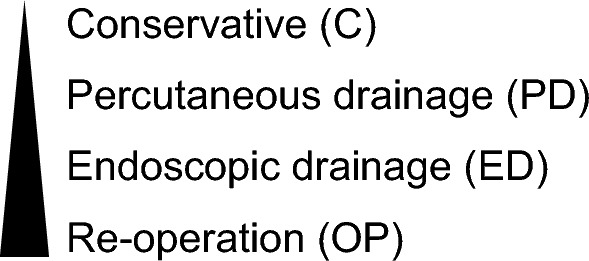



#### Comparative analyses (results “Clinical resolution of anastomotic leakage after treatment”)

Patients were grouped in a stratified, decision-based algorithm, in which primary-, secondary- and tertiary interventions were defined. The algorithm allowed us to directly compare the technical modalities at given phases in sometimes alternating sequences of treatment modalities during the post-operative clinical course:

(i) Primary intervention–First intervention after diagnosis of peri-anastomotic fluid collection and/or anastomotic leakage
(ii) Secondary intervention–Post-procedural intervention after primary intervention
(iii) Tertiary intervention–Post-procedural intervention after secondary intervention

The primary endpoints of the study were hospitalization (length of hospital stay) and clinical success (treatment success rate, primary/secondary resolution) of interventional treatment modalities. We defined clinical resolution as a sub-clinical, ambulatory situation without further need for interventions, and antibiotics, while maintaining normal mobility and food intake (= day of discharge). Therefore, clinical resolution directly correlated with in-hospital stay. However, due to often alternating treatment modalities (range: 0–4) for diagnosed anastomotic leakage, we created the decision-based algorithm (*comparative analyses*). Secondary endpoints considered post-interventional infectious and drainage parameters, morbidity/mortality and disease-specific cumulative survival.

### Peri-anastomotic drainage technique

In cases of clinical and radiographic suspicion of a peri-anastomotic leakage, we inspected the anastomosis through direct endoscopy. Anastomotic leakage could typically be identified by pus secretion or direct visualization, without the need of endoscopy-guided ultrasound (EUS). Following contrast medium injection and simultaneous fluoroscopy, we used a guidewire to place double-pigtail stents peri-anastomotic. If direct endoscopy did not visualize a dehiscence or size did not match with pre-interventional radiologic findings, the use of additional EUS confirmed (or excluded) any perianastomotic fistula with fluid collection, which was subsequently drained by the internalized drainage in the technique described previously [15]. In cases of larger dehiscence and necrotic material, we performed additional endoscopic necrosectomy. The size of dehiscence determined the number of stents used (range: 1–5). In cases of drained fluid collections, aspirates underwent microbiologic analyses. Once internal drainages were in place, external drainages were removed, generally at the same day (internalization). We scheduled endoscopic removal of internal stents around 6 weeks after discharge.

### Statistics

For the data analyses, we used the statistical software package SPSS (IBM Corp. Released 2021. IBM SPSS Statistic, Version 28.0. Armonk) and visualized using Prism (GraphPad Software, La Jolla California USA). For descriptive analyses of patients’ demographics, we performed one-way Anova for continuous and Fischer’s exact for categorical variables. We applied ordinal logistic regression to determine independent predictors of complication management. For comparative outcome analyses and follow-up of treatment groups, Cox-regression was used, controlling for underlying disease and tumor stage. Kaplan-Mayer regression was used to compare clinical resolution.

## Results

### Cohort characterization

During the study period from 2015 to 2020 a total of 795 pancreatic resections were performed. Of those, 531 patients (66.8%) underwent pancreatoduodenectomies (PPPD/Whipple), 216 left-sided pancreatectomies (27.2%) and 48 patients (6.0%) had other surgical procedures (total pancreatectomy, central pancreatectomy, enucleation etc.). Following PPPD/Whipple, 403 patients (73%) received pancreato-gastric reconstruction of which 110 patients (27.3%) fulfilled inclusion criteria for comparative evaluation of treatment approaches for either anastomotic leakage and/or pancreatic fistula. Most patients received a PD (*n* = 50; 45.5%) as a single interventional treatment modality while around 16.4% (*n* = 18) were sufficiently treated through conservative concepts. Close to one-third (n = 32; 29.1%) of our patients were treated with peri-anastomotic pigtail stents (ED) for internal drainage of peri-anastomotic fluid collections as primary-, secondary-, and/or tertiary treatment modality. Ultimately, 12 patients (10.7%) needed re-operation (OP). Patient characteristics of weighted treatment groups are listed in Table 1. Statistical comparison on patient demographics were primarily conducted on interventional (PD, ED, OP) groups. The latter identified sex as single significant parameter, likely due to uneven distribution already present in the baseline characteristics of our cohort (76 male vs 34 female). In addition, pre-treatment conditions (BMI, previous abdominal surgery, ASA) and peri-operative variables (operative time, surgical technique) were not predictive of treatment allocation in ordinal logistic regression analyses (Table 2).

**Table 1:** Postoperative Management after PPPD with clinically relevant perianastomotic fluid collection

	Treatment groups*								
	Conservative (C)	%	Percutanous drain (PD)	%	Endoscopic drain (ED)	%	Re-operation (OP)	%	Statistics (*p*)**
Total cases	18	16.3	50	45.5	30	27.3	12	10.9	
Age	64.1 (± 10.1)		63.9 (± 12.4)		67.0 (± 9.2)		62.3 (± 13.1)		*0.76*
Sex	M 14 W 4	77.8 22.2	M 29 W 21	58 42	M 26 W 4	86.7 13.3	M 7 W 5	58.3 41.7	*0.05*
Body mass index (BMI)	25,7 (± 4.9)		25.2 (± 4.4)		26,2 (± 6.6)		27.1 (± 6.2)		*0.89*
ASA	ASA1: 1 ASA2: 9 ASA3: 8	5.6 50.0 44.4	ASA1 2 ASA2 30 ASA3 17	4.1 61.2 34.7	ASA1 0 ASA2 16 ASA3 13	0 55.2 44.8	ASA1 1 ASA2 7 ASA3 4	8.3 58.3 33.3	*0.69*
Surgery technique	Open: 14 Robotic: 4	77.8 22.2	Open: 45 Robotic: 5	90.0 10.0	Open: 22 Robotic: 8	73.3 26.7	Open: 7 Robotic: 5	58.3 41.7	*0.12*
Previous abdominal surgery	No: 12 Yes: 6	66.7 33.3	No: 33 Yes: 17	66.0 34.0	No: 7 Yes: 23	19.4 80.6	No: 5 Yes: 7	41.7 58.3	*0.13*
Operation time (min)	347.2 (± 68.2)		353.8 (± 84.3)		289.1 (± 59.0)		331.7 (± 59.5)		*0.06*
Surgery indication	Benign: 6 Malign: 12	33.3 66.7	Benign: 13 Malign: 37	26.0 74.0	Benign: 7 Malign: 24	23.3 76.7	Benign: 4 Malign: 8	33.3 66.7	*0.39*
Tumor entity	PDAC: 7	38.9	PDAC: 17	34.0	PDAC: 11	36.7	PDAC: 5	41.7	
	Duodenal/Papillary: 2	11.1	Duodenal/Papillary: 3	6.0	Duodenal/Papillary: 3	10.0	Duodenal/Papillary: 1	8.3	
	Ampullary: 1 Distal bile duct: 2 Other: 6	5.6 11.1 33.3	Ampullary: 5 Distal bile duct: 8 Other: 17	10.0 16.0 34.0	Ampullary: 1 Distal bile duct: 6 Other: 9	3.3 20.0 30.0	Ampullary: 1 Distal bile duct: 1 Other: 4	8.3 8.3 33.3	*0.62*

*Group assignment depending on most invasive treatment during course of hospitalization (step-up approach)

**ANOVA for continous variables or Fisher-Exact test for categorical variables

**Table 2:** Ordinal logistic regression analysis of pre-operative and peri-operative variables

Variable	Odds ratio	Estimate	95% Confidence interval		*p*-value
			Lower	Upper	
Sex (female vs male)	0.794	−0.231	0.321	1.963	0.617
Body mass index (BMI)	1.145	0.135	0.405	3.236	0.798
Age	0.997	−0.001	0.963	1.038	0.977
ASA (ASA 1/2 vs 3)	1.191	0.175	0.544	2.608	0.661
Diabetes mellitus II	0.955	−0.046	0.367	2.487	0.925
Smoking	0.955	−0.157	0.443	1.647	0.639
Alcohol	1.091	0.087	0.525	2.269	0.815
Past abdominal surgery	1.173	0.159	0.468	2.940	0.734
Tumor entity (malign vs benign)	1.297	0.260	0.281	5.983	0.739
T stage (T3/4 vs T1/2)	0.931	−0.072	0.560	1.548	0.782
Operation technique (robotic vs open)	1.513	0.414	0.895	2.557	0.122
Operation time	0.996	−0.004	0.992	1.000	0.066

Dependent variable: Ascending treatment groups in terms of invasiviness

### Treatment course of patients with anastomotic leakage after pancreatogastrostomy

**Fig. 1:**
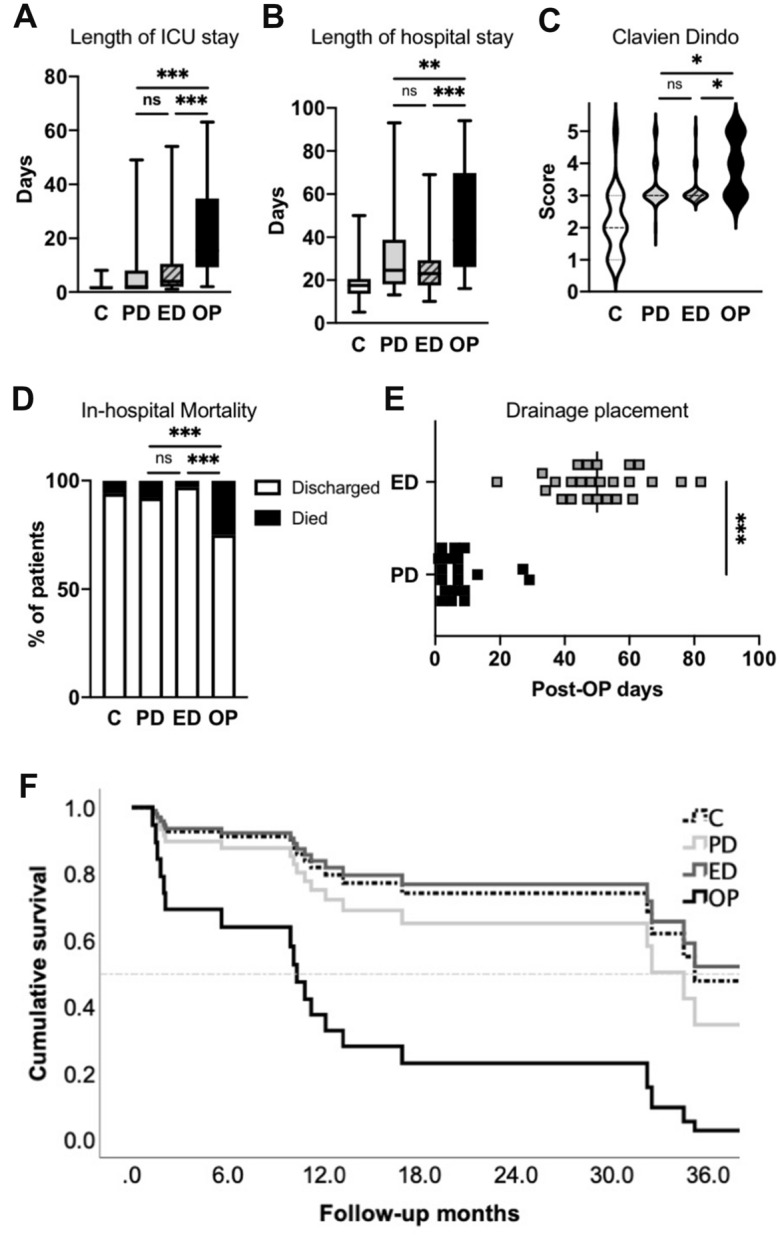
**A**–**D** Comparative analyses of weighted treatment groups for length of ICU stay, length of hospital stay, Clavien-Dindo classification and in-hospital mortality (one-way ANOVA with Bonferroni multiple comparison). **E** Comparative analyses of ED- and PD treatment groups for total drainage placement in-situ (Welch’s unpaired *t*-test). **F** Analyses of cumulative survival (Cox-Proportional-Regression) of treatment groups, controlling for underlying disease

Following the pancreatoduodenectomy, all patients were admitted to the ICU (100%). Most patients were transferred to regular wards at postoperative day 1 (42.7%; median 2.0). On average, the timing of complication (anastomotic leakage) occurred at day 7.5 (± 4.6 days; range: 1–25 days) discovered either by CT-scan or endoscopic validation. Postoperative parameters as surrogates for systemic inflammation (CRP/Leukocytes) averaged 155.4 mg/l (± 96.2 mg/l; range 10–467.8 mg/l) and 14.5/nl (± 5.9/nl; range 3.8–38.9/nl) at day of diagnosis. Drainage lipase values were 18,052.4 U/l (± 48,217.2 U/l; range 3–327,687 U/l) at time of diagnosis. Treatment started at postoperative day 9.5 (± 5.9 days) for PD, while ED treatment was performed at postoperative day 14.5 (± 6.4 days; p < 0.01). ICU re-admission was needed in 11 cases (22%) for PD- vs. 12 cases (38.7%) for ED group (*p* = 0.15, Fisher´s Exact). Total duration (days) on ICU of respective treatment groups is shown in Fig. 1A. Length of in-hospital stay was 19.4 days for C (± 10.9 days), 30.5 days (± 16.1 days) for PD, 25.0 days (± 11.4 days) for ED and 47.3 for OP (± 26.9 days) (Fig. 1B , *p*< 0.001, one-sided ANOVA). Overall complications in treatment groups were documented according to classifications of Clavien-Dindo (Fig. 1C) and the ISGPS society (Table 3). In-hospital mortality of primary interventions was significantly reduced in the interventional groups (PD, ED) compared to re-operation (Fig. 1D). Comparing the total drainage placement of interventional groups, drainage remained significantly longer in ED compared to PD group (Fig. 1E), due to varying treatment standards. Cox-Proportional-Regression analyses on patients with malignant diagnosis (PDAC, ampullary cancer, papillary cancer, distal bile duct cancer) revealed different cumulative survival in weighted treatment groups, showing reduced survival for patients who needed re-operation (Fig. 1F; Table 4).

**Table 3:** Post-operative complications after pancreatic surgery (ISGPS definition of 2016)

	Treatment groups									
	Conservative (C)	%	Percutanous drain (PD)	%	Endoscopic drain (ED)	%	Re-operation (OP)	%	Statistics*	Statistics**
Total cases	18	16.4	50	45.5	30	27.3	12	10.9		
Pancreatic fistula (POPF)	A: 18 B: 0 C: 0	100 0 0	A: 0 B: 50 C: 0	2 98 0	A: 0 B: 30 C: 0	0 100 0	A: 0 B: 0 C: 12	0 0 100	< *0.001*	*NA*
Hemorrhage (PPH)	None: 16 A: 0 B: 1 C: 1	88.9 0 5.6 5.6	None: 43 A: 1 B: 3 C: 3	86.0 2 6.0 6.0	None: 23 A: 0 B: 6 C: 1	76.7 0 20 3.3	None: 3 A: 0 B: 1 C: 8	25 0 8.3 66.7	< *0.001*	*0.24*
Surgical site infections (SSI)	None: 16 1/2: 2 3: 0	88.9 11.1 0	None: 0 1/2: 0 3: 50	0 0 100	None: 0 1/2: 0 3: 30	0 0 100	None: 1 1/2: 0 3: 11	8.3 0 91.7	< *0.001*	*NA*
Deleayed gastric emptying (DGE)	None: 15 Yes: 3	83.3 16.7	None: 36 Yes: 14	72.0 28.0	None: 28 Yes: 2	93.3 6.7	None: 8 Yes: 4	66.7 33.3	*0.09*	*0.16*

*NA* Not applicable

*Chi^2^ test for categorical variables (all groups)

**Fisher’s exact test for categorical variables (PD vs ED)

**Table 4:** Cox-regression overall survival analysis of patients diagnosed with PDAC

Variable	Hazard ratio*	Standard error	Estimate	95% Confidence Interval		*p*-value
				lower	upper	
Conservative (C)			*6.738*			0.081
Percutanous Drain (PD)	1.435	0.615	0.346	0.430	4.788	0.557
Endoscopic Drain (ED)	0.882	0.795	0.025	0.186	4.195	0.875
Re-Operation (OP)	4.896	0.739	4.617	1.150	20.847	0.032

*Hazard calculated with conservative group as indicator

### Clinical resolution of anastomotic leakage after treatment

**Fig. 2:**
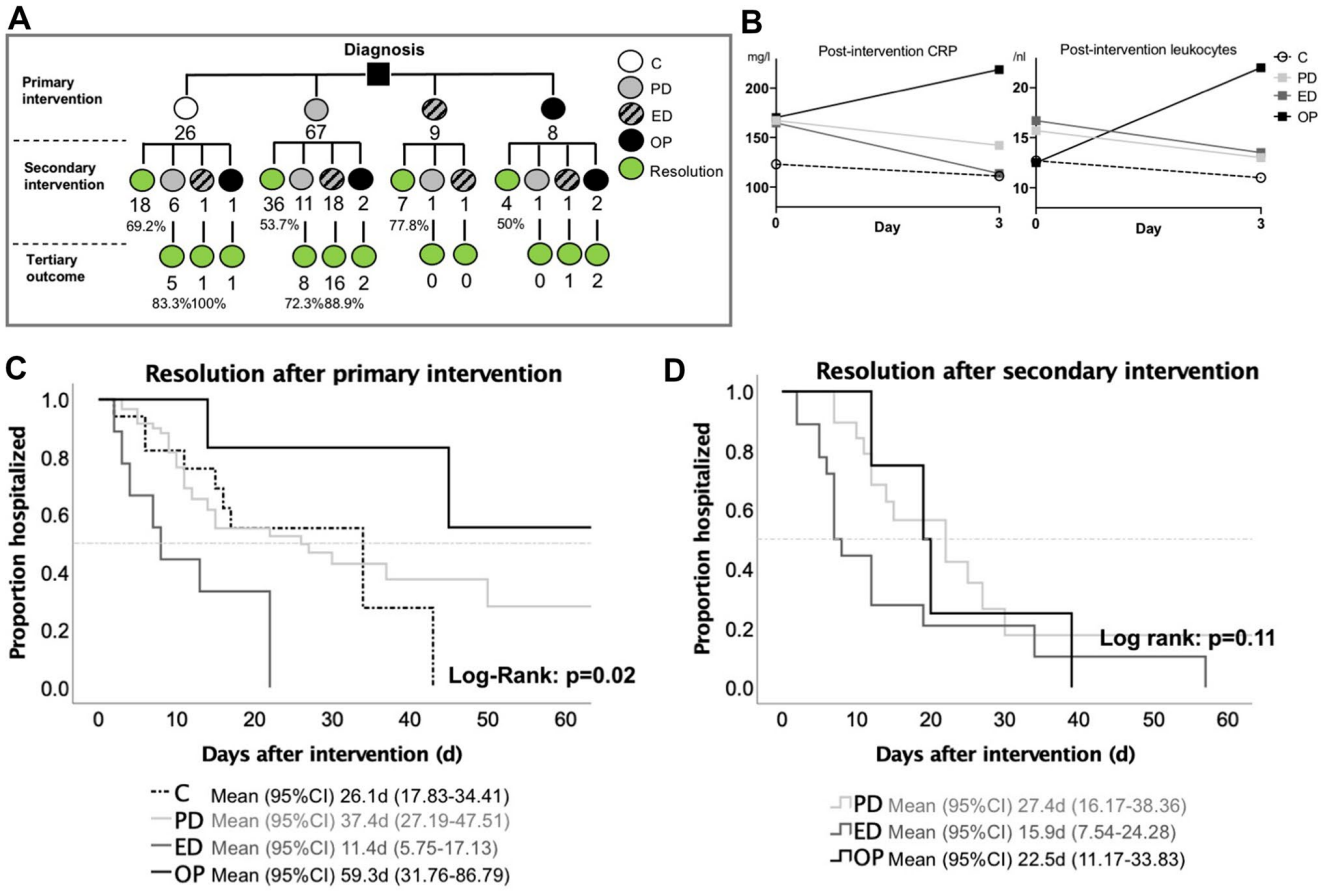
**A** Decision tree of patients, regularly receiving combinatory treatments (primary-/secondary-/tertiary treatment) for anastomotic leakage and/or peri-anastomotic fluid collection. Below annotated, the number of patients and success rates of respective sub-groups. **B** Post-procedural development of infectious parameters. **C**–**D**. Kaplan-Maier analyses of clinical resolution after the primary- and secondary intervention

Primary success rate, meaning that the first intervention led to resolution, varied between groups (Fig. 2A). On average, we achieved primary success in the conservative group in 69.2%, in the PD group in 53.7%, in the ED group in 77.8% and in the OP group in 50.0% (*p* = 0.58, Fischer´s Exact). A direct comparison of the interventional groups PD and ED indicated no significant difference (*p* = 0.16, Fischer´s Exact). Following the primary intervention, inflammatory parameters (CRP/Leukocytes) at post-intervention day 3 immediately decreased in the interventional groups (PD, ED) while parameters in the OP group remained high (Fig. 2B). Comparing inflammatory parameters at post-intervention day 3 in both, primary- and secondary intervention settings, showed a tendency for decreased infectious parameter in the ED as compared to the PD group (Table 5). Analyzing the clinical resolution of primary interventions, we observed earlier resolution in the ED group, compared to the PD- and OP group which reaches statistical significance (*p* = 0.02, Log-rank test; Fig. 2C). A secondary modality for anastomotic leakage was required in eight cases (30.8%) for the conservative-, 31 cases (46.3%) for the PD-, two cases (22.2%) for the ED- and in four cases (50%) for the OP group (Fig. 1). Following secondary treatment, success rates with interventional groups were 68.4% in the secondary PD group, 85.7% in the secondary ED group and 100% in the secondary OP group, respectively. Kaplan-Maier analyses on resolution after secondary intervention only characterized a tendency towards reduction in the ED group compared to the PD group (*p* = 0.11, Log-rank test**,** Fig. 2D). In summary, treatment stratification into decision steps, discriminated advantages of ED over PD treatment. Thus, in some observations (inflammatory parameters, resolution after primary intervention) endoscopic treatment outperformed percutaneous intervention.

**Table 5:** Infectious parameters after interventions

Intervention group	Post-intervention (d3)* Primary intervention											Post-intervention (d3)* Secondary intervention										
	n	CRP (mg/l)	STD	Range	Statistics**	Post-hoc***	Leukocytes (/nl)	STD	Range	Statistics**	Post-hoc***	n	CRP (mg/l)	STD	Range	Statistics**	Post-hoc***	Leukocytes (/nl)	STD	Range	Statistics**	Post-hoc***
Conservative (C)	18	111.2	± 71.9	20.3–315.3	0.018		11.1	± 4.0	5.0–18.7	< 0.001		–	–	–	–	0.015		–	–	–	< 0.001	
Percutanous Drain (PD)	67	141.9	± 81.5	18.5–444.4		ns	13.1	± 5.6	4.0–33.7		ns	19	108.2	± 36.1	36.2–87.3		ns	12.4	± 6.3	9.3–15.4		ns
Endoscopic Drain (ED)	9	113.7	± 69.9	39–0-234.9			13.7	± 5.5	6.8–25.8			20	91.4	± 93.3	7.5–323.6			10.7	± 5.7	7.9–13.4		
Re-Operation (OP)	8	218.8	± 114.4	60.3–345.6			21.9	± 13.3	6.0–39.5			5	215.3	± 123.1	42.9–271.5			25.4	± 13.8	8.2–42.5		

* Laboratory results at day of intervention (± 1d)

** One-sided ANOVA

*** Bonferroni multiple comparisons

### Endoscopic drainage

**Fig. 3:**
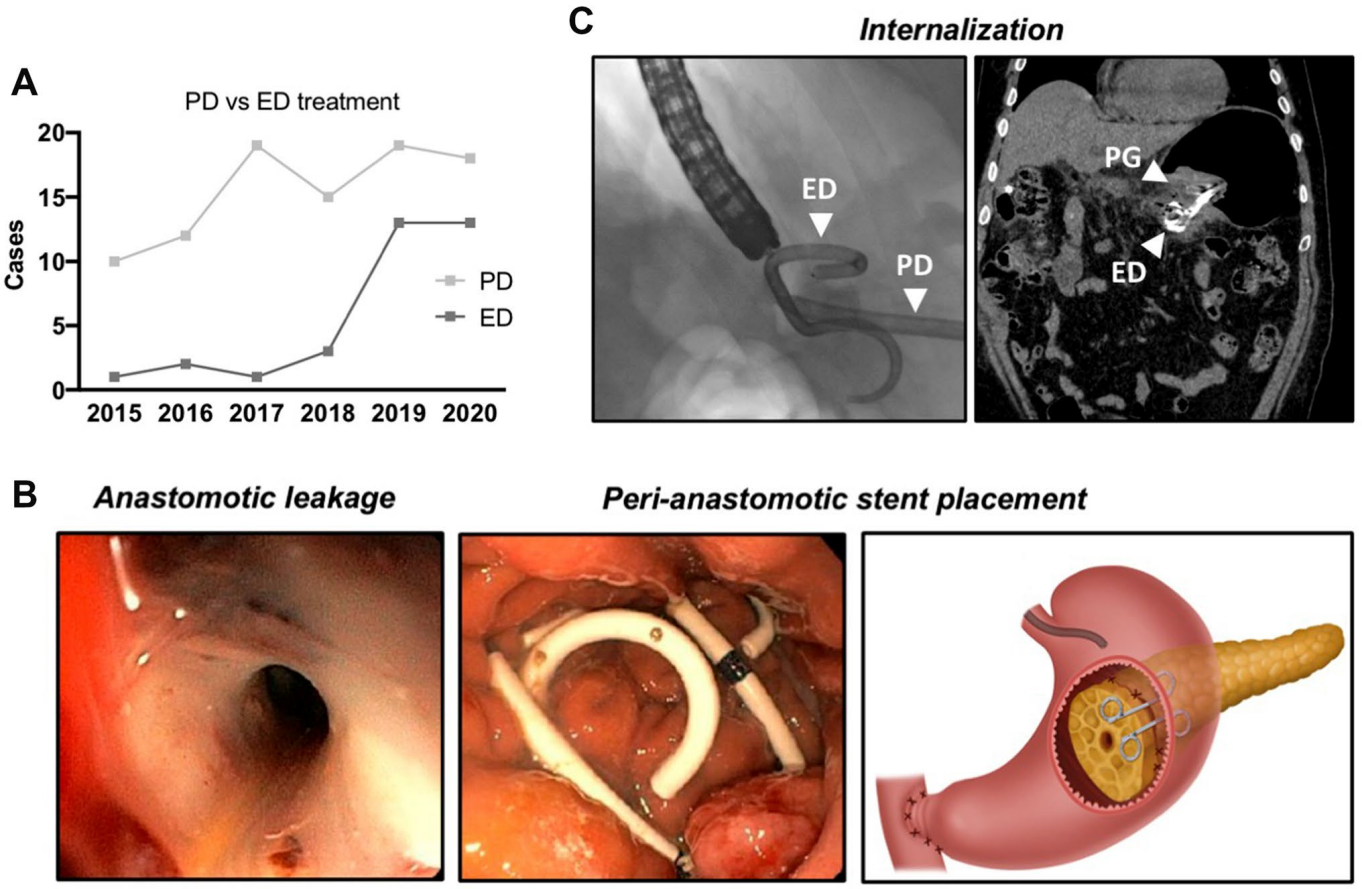
**A** The number of endoscopic treatments over the years 2015–2020. **B** Representative pictures during endoscopic intervention showing large dehiscence of the PG with subsequent placement of two plastic pigtail stents. **C** A guidewire and the tip of the percutaneous drainage (PD) helped to correctly place the peri-anastomotic pigtail stents (ED). Representative post-interventional CT-scan shows correct stent placement and internal drainage of peri-anastomotic fluid collection at the pancreato-gastrostomy (PG)

In 29 patients ED was performed either as a primary or secondary treatment modality (three cases as tertiary intervention). Based on positive experiences with the treatment of fluid collections after distal pancreatectomy, we expanded and increasingly applied the technique to patients after pancreatoduodenectomy with pancreato-gastrostomy (PG) (Fig. 3A). Facilitated monitoring after surgery by endoscopic inspection reflects the inherent advantage of applying the PG reconstruction. In cases of confirmed anastomotic leakage with subsequent endoscopic treatment, we placed at least one plastic pigtail stent (median 1, range: 1–5) right across the dehiscence (Fig. 3B). On average a single (median 1, range: 1–2) intervention sufficed (technical success rate of 93.3%). EUS was only needed in 31.3% (10 out of 32 cases) for assessment of peri-anastomotic fluid collection. The percutaneous drainage was sometimes used as a landmark for correct stent placement but was generally removed after the procedure (internalization) (Fig. 3C). In a few cases with larger dehiscence and abscess formation with mature walls, we additionally performed necrosectomy prior to stent placement (6 out of 32 cases). Adverse events directly linked to endoscopic intervention were not documented during the observational period (complication rate: 0%). Endoscopic re-interventions for stent correction were needed in two cases (6.3%). On average, drainages were electively removed around 6 weeks after discharge (54.1 postoperative days; ± 28.2 days). Variances occurred due to the different clinical courses of included individuals. Incidental stent migration, detected in routine X-rays prior to second intervention, occurred in seven patients (21.8%) and were not associated with any clinical deterioration.

## Discussion

Across pancreatic cancer surgery centers post-operative fistula and anastomotic leakage are widely regarded the Achilles’ heel [18]. We have learned that complication management is key for decreasing morbidity and mortality after pancreatic resection [3, 19]. Through institutional experience as a high-volume center, we to further advanced on interdisciplinary concepts for treating pancreatic fistulas and anastomotic leakage. This study demonstrates the feasibility, efficiency, and safe application of an endoscopic technique with the placement of peri-anastomotic stents for internal drainage of peri-anastomotic fluid collection after pancreatoduodenectomy. Consequently, we demonstrate earlier resolution and reduced hospitalization of patients with anastomotic leakage, better aligning with modern ERAS concepts that ultimately allow for earlier admission of oncologic patients to adjuvant treatments.

EUS-guided concepts for the drainage of peri-luminal fluid collections are not new and have been tested for some time. The main indications remain in the treatment of digestive fluids after pancreatic surgery for preventing severe complications. Such techniques have been initially conducted in pseudocysts or peri-gastric fluid collections after distal pancreatectomies, however focusing on late abscess formation with mature walls [7, 8, 20]. We and others have shown that the trans-gastric route allows for uncomplicated and safe internal drainage even in the absence of abscess formation for early post-operative treatment of clinically relevant pancreatic fistulas [15, 21]. Compared to percutaneous interventions, success rates revealed advantages of transmural drainage systems in some settings [7, 22]. Positive experiences have also been documented for other indications, including bariatric surgery and liver resections [10, 23].

In our series, we routinely performed reconstruction with pancreato-gastric anastomosis after pancreatoduodenectomy (Whipple procedure/PPPD) [16, 17]. This surgical technique facilitates early monitoring of pancreatic fistulas and anastomotic leakage, using contrast agents through located nasogastric tubes and/or endoscopic intervention. In cases with detectable dehiscence, early stent placement was conducted (on average at POD 14.5) and demonstrated primary success rates of 77.8% (7/9 patients) while this was documented in only 53.7% (36/67 patients) of cases with percutaneous drainage. Also in the setting of secondary intervention, endoscopic drainage showed advantages over percutaneous management (85.7% in ED groups vs. 68.4% in PD group). Despite limited sample sizes, our study adds to accumulating evidence of using transmural over percutaneous interventions as preferred treatment for peri-anastomotic fluid collections and pancreatic fistula. At a minimum, such approaches should be widely established as a versatile and safe adjunct to conventional percutaneous techniques. Future studies with larger patient cohorts may define algorithms and characteristics of which patients most likely benefit from either of these treatment modalities.

The rationale for internalizing external drainages lies in the aim of earlier recovery, reduced hospitalization and an improved quality of life. Although it remains difficult to accurately define resolution, studies could show similar or even reduced hospital stay in cases with endoscopic stent placement when compared to percutaneous techniques [7, 24]. This was also a conclusion of a recent meta-analysis even when certainty of evidence remains limited overall [25]. In our study, we examine advantages of earlier resolution via ED compared to PD in the setting of primary intervention. Similar tendencies were observed in the setting of secondary interventions, not reaching statistical significance however, likely due to the patients’ overall more complicated clinical courses. Also, laboratory surrogates suggested more effective fistula treatment of ED over PD in both, primary and secondary interventions. Despite limited sample sizes, we are confident that our preliminary observation of reduced hospitalization through internalization can be confirmed when using a stratified, decision-based algorithm for comparative analyses.

Several studies reported adverse events or complications after endoscopic stent placement [21, 25]. However, we were not able to directly assign complications to the endoscopic treatment, which may be routed in our technique which uses a pre-existent dehiscence, not puncturing the gastro-intestinal wall.

Data on experiences of transmural drainage after pancreatoduodenectomy are scarce, possibly due to the often distant location of fluid collections in the peri-hepatic cavity after pancreato-jejunostomy. Tilara A et al. reported nine patients after pancreatoduodenectomy, while using a trans-gastric route and not documenting specific success rates for this cohort [9]. Al-Efishat M et al*.* demonstrated similar success rates and outcomes between ED and PD in a matched-pair analyses, ultimately including 20 patients after pancreatoduodenectomy, again using procedures puncturing the intestinal wall [11]. Our study is unique, in that we include a large cohort of patients treated with an innovative approach using peri-anastomotic stents in the setting of pancreato-gastric reconstruction after pancreatoduodenectomy. This combination enabled a standardized interdisciplinary work-flow between surgeons, radiologists and endoscopists with measurable benefits for our patients suffering from complications such as anastomotic leakage and pancreatic fistula.

As a retrospective study, we cannot overcome the inherent selection bias. During the study period 2015–2020, there was an increase of patients selected for our adopted technique due to positive experiences, while the percutaneous technique were more prevalent at earlier time points. Overall, sample sizes of conceptional groups were limited, such that statistical analyses is challenged and therefore need to be interpreted with caution. We seek to collect prospective data in order to obtained more robust analyses on these treatment modalities. In addition, this study was performed at a single institution, due to the project-related demands of a specific surgical technique (pancreatogastrostomy) in combination with excellent endoscopic expertise for ultrasonography-guided intervention. Challenges also lay in the often heterogenous and individual approaches of complication management, which we attempted to address by analyzing cohorts with a stratified, decision-based algorithm (primary, secondary, tertiary interventions) while being aware of its limitation to control for other more complicated confounders. Lastly, decisions were sometimes based on individual clinical preferences not always allowing for standardized, clinical algorithms.

This study confirms the importance of endoscopy-guided approaches as a primary treatment or adjunct procedure to address complications after pancreatoduodenectomy. We herein report a novel, innovative technique for internal drainage of peri-anastomotic fluid collections in the setting of pancreato-gastric reconstructions.
